# Bibliometric Analysis of the Scientific Literature on Rheumatoid Arthritis-Associated Interstitial Lung Disease

**DOI:** 10.1155/2021/7899929

**Published:** 2021-12-20

**Authors:** Yuan Zhang, Tingxiao Zhao, Tianjin Wu, Wei Huang, Teng Wu, Yunjuan Shi, Zhenhua Ying

**Affiliations:** ^1^Zhejiang Provincial People's Hospital, Hangzhou, Zhejiang, China; ^2^Hangzhou Medical College People's Hospital, Hangzhou, Zhejiang, China; ^3^Bengbu Medical College, Bengbu, Anhui, China; ^4^Zhejiang Chinese Medical University, Hangzhou, Zhejiang, China; ^5^Qingdao University, Qingdao, Shandong, China

## Abstract

**Background:**

In recent years, the number of studies on rheumatoid arthritis-related interstitial lung disease (RA-ILD) has been increasing, which has led to many publications on this topic. Our purpose is to identify research trends in RA-ILD and analyze the most-cited RA-ILD-related high-quality scientific publications.

**Methods:**

All publications on RA-ILD in the Core Collection database of Web of Science were searched. The publication year, country, institution, total citations, and journal were extracted and analyzed. We used VOSviewer software or an online bibliometric analysis platform for cooccurrence analysis of the keywords, institutions, and countries involved. The 100 most frequently cited RA-ILD publications were analyzed.

**Results:**

In total, 596 publications related to RA-ILD were obtained. Over time, the frequency of RA-ILD publications has increased. Globally, the United States provides the most publications on RA-ILD (*n* = 195). The institution with the highest publication output was the Mayo Clinic (*n* = 43). The journal “Annals of the Rheumatic Diseases” published most with 93 articles and received 338 citations. A clinical description was the most common research topic in RA-ILD-related publications.

**Conclusions:**

In recent years, there has been an increasing number of studies on RA-ILD, and related publications have increased rapidly. This study is the first bibliometric study of RA-ILD-related publications. It can be used as a guide for clinicians and can help researchers choose research directions of interest in this field.

## 1. Introduction

Rheumatoid arthritis (RA) is a systemic autoimmune disease characterized by articular and extra-articular manifestations affecting approximately 1-2% of the general population [1]. Interstitial lung disease (ILD) is an extra-articular manifestation of RA, which occurs frequently in up to 80% of patients with RA. This may be the result of chronic immune activation and inflammation in RA, or the pulmonary toxicity caused by immunomodulatory drugs used to treat RA [2–6]. The prevalence of RA-ILD ranges from 1% to 58%, depending on the diagnostic means used and the severity in the RA population studied [6, 7]. Currently, ILD is the second leading cause of death in patients with RA after cardiovascular disease [8]. Research on RA-ILD has increased to include its natural history, pathogenesis, radiological evaluation, clinical manifestations, and treatment [9–12]. However, the trend of RA-ILD research is unclear, and the most influential research in this field has not been systematically determined. Therefore, our purpose was to provide a bibliometric study of publications on RA-ILD.

Bibliometric analysis is a convenient and reliable statistical method that can quantitatively and qualitatively evaluate research trends in the research field. This analysis has long been used in the field of medical research and has been widely accepted by scientific researchers [13–15]. To the best of our knowledge, no bibliometric studies on RA-ILD have been published to date. Therefore, in this study, we use bibliometric statistical methods to identify the most influential publications and analyze the research status and trends in the RA-ILD research field.

## 2. Materials and Methods

### 2.1. Datasource

All the data of this study were obtained from articles retrieved from the core collection database of Web of Science on July 1, 2021.

### 2.2. Search Strategy

The retrieval steps and strategies were as follows: Title = rheumatoid arthritis AND Title = (interstitial lung disease OR interstitial pneumonia) AND Language = English AND Document type = (review OR article) AND Time span =1980 to 2021.

### 2.3. Statistical Tools

VOSviewer, an online bibliometric analysis platform (https://bibliometric.com), and Excel software were used to extract and analyze all data. VOSviewer is a software that is usually used to visually analyze the collaborative network between countries, institutions, and authors and cocitation of keyword clusters to analyze research trends and hotspots. The role of the online bibliometric analysis platform is similar to that of VOSviewer. Excel software was used to extract and analyze various details of the publication, including author, title, journal, year of publication, institution, country, journal impact factors, and number of total citations.

### 2.4. Data Extraction

According to the retrieval steps and strategies, the two authors independently fetched the article information and discussed the differences until they reached a consensus. Data were obtained from the core collection database of Web of Science, and the publication information was extracted and analyzed using Excel, online bibliometric analysis, and VOSviewer software.

## 3. Results

### 3.1. Publication Analysis

**Figure 1:**
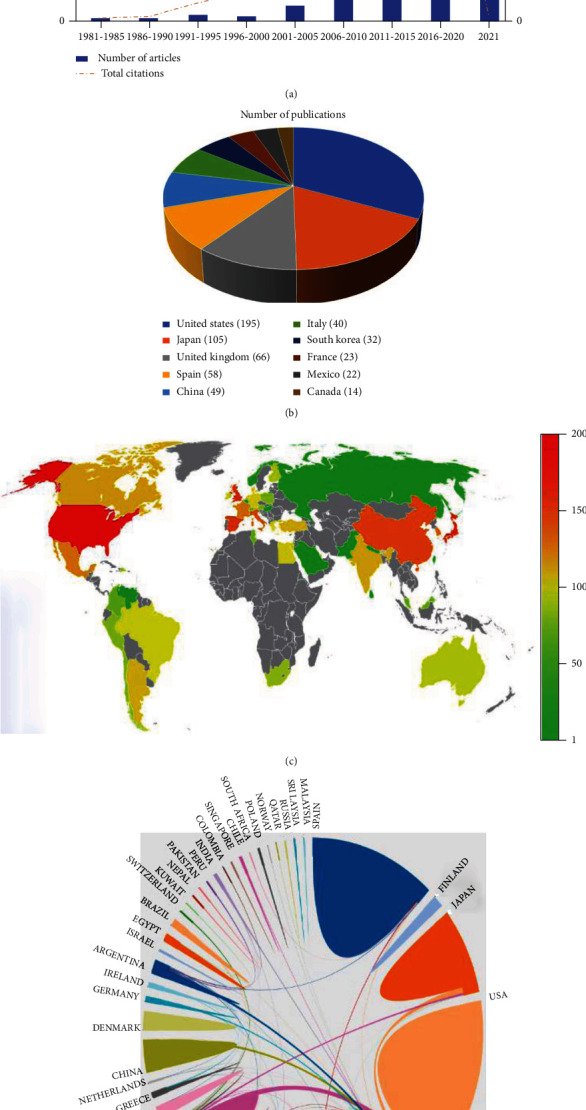
Overview of publications. (a) Number of publications and citations from 1981 to 2021. (b) Sources of publications. (c) Top 10 countries. (d) International collaborations.

A total of 596 RA-ILD research articles were found in the core collection database of the Web of Science. The number of articles increased from 1981 to 2021 (Figure 1(a)). Quantitative analysis shows that in the past 10 years, global research on RA-ILD has increased rapidly, from four articles from 1981 to 1985 to 326 articles from 2016 to 2020. This result shows that RA-ILD has attracted increasing attention, and the research process of RA-ILD continues to accelerate.

### 3.2. Countries Analysis

These articles cover 46 countries and regions. Globally, the United States (US) published the most studies (*n* = 195), followed by Japan (*n* = 105), the United Kingdom (*n* = 66), Spain (*n* = 58), China (*n* = 49), Italy (*n* = 40), South Korea (*n* = 32), France (*n* = 23), Mexico (*n* = 22), and Canada (*n* = 14) (Figures 1(b) and 1(c)).

The online bibliometric analysis platform was used to analyze cooperative relations between countries. The visual analysis shows that the USA has always been the center of RA-ILD research in the world, and Japan, France, China, and South Korea have been found to be potential research powers. (Figure 1(d)).

### 3.3. Institutions Analysis

**Figure 2:**
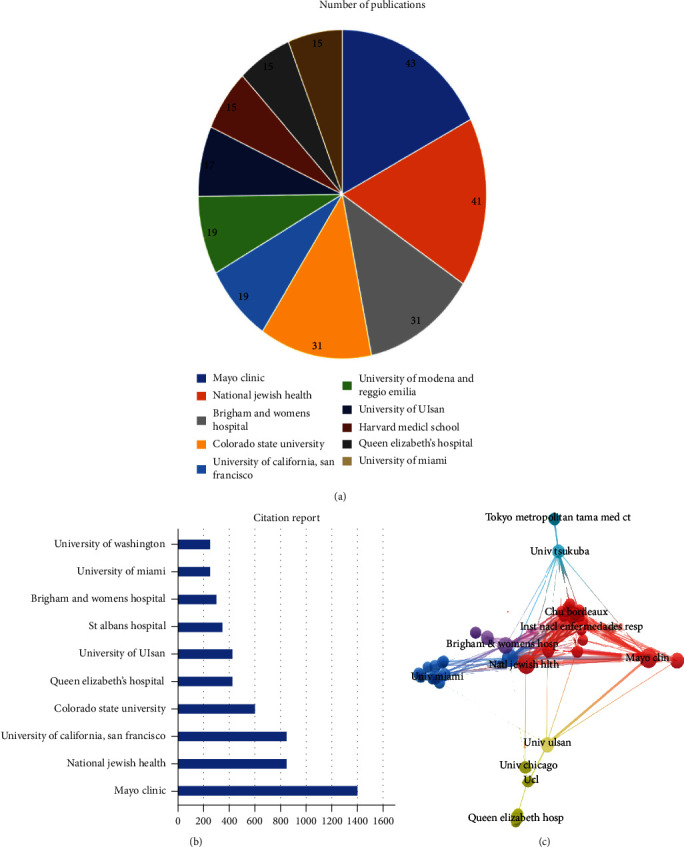
Highest impact institutions. (a) The top 10 institutions (publications). (b) The top 10 institutions (citations). (c) Institutional collaborations.

All the publications involve 1000 institutions. The results show that many institutions in the United States actively participate in RA-ILD research. The 10 most productive institutions internationally were Mayo Clinic (*n* = 43), National Jewish Health (*n* = 41), Brigham and Women's Hospital (*n* = 31), Colorado State University (*n* = 31), University of California, San Francisco (*n* = 19), University of Ulsan (*n* = 19), University of Modena and Reggio Emilia (*n* = 17), Harvard Medical School (*n* = 15), Queen Elizabeth's Hospital (*n* = 15), and University of Miami (*n* = 15), respectively (Figure 2(a)). According to the citation report, Mayo Clinic's articles were cited the most, namely, 1408 times, followed by the National Jewish Health, which was cited 858 times, and the University of California, San Francisco, 851 (Figure 2(b)).

VOSviewer software was used to analyze the extent of cooperative relations between institutions. The institution with the most links, i.e., the highest link strength was recorded by the National Jewish Health Organization (*n* = 131), followed by the University of Colorado (*n* = 123), Mayo Clinic (*n* = 119), and Brigham and Women's Hospital (*n* = 117). In the VOSviewer software, the width of the line reflects the close relationship of interinstitution cooperation. The National Jewish Health had close collaborations with the University of Colorado and Mayo Clinic. Mayo Clinic had large collaborations with National Jewish Health, University of Colorado, Brigham and Women's Hospital, and Harvard Medical School (Figure 2(c)).

### 3.4. Journals Analysis

All 596 articles in this study were published in 123 journals. Among these, the journals which had published at least 20 articles on the topic accounted for 73.8% of the total (Table 1). The five journals with the most articles on the topic were Annals of the Rheumatic Diseases, Arthritis & Rheumatology, American Journal of Respiratory and Critical Care Medicine, Rheumatology, and Arthritis and Rheumatism. Moreover, articles in the Annals of the Rheumatic Diseases have been cited the most. More than 20 journals published on RA-ILD, and the average impact factor was 11.8, indicating a high level of reliability of the included studies.

### 3.5. Research Status and Analysis

**Figure 3:**
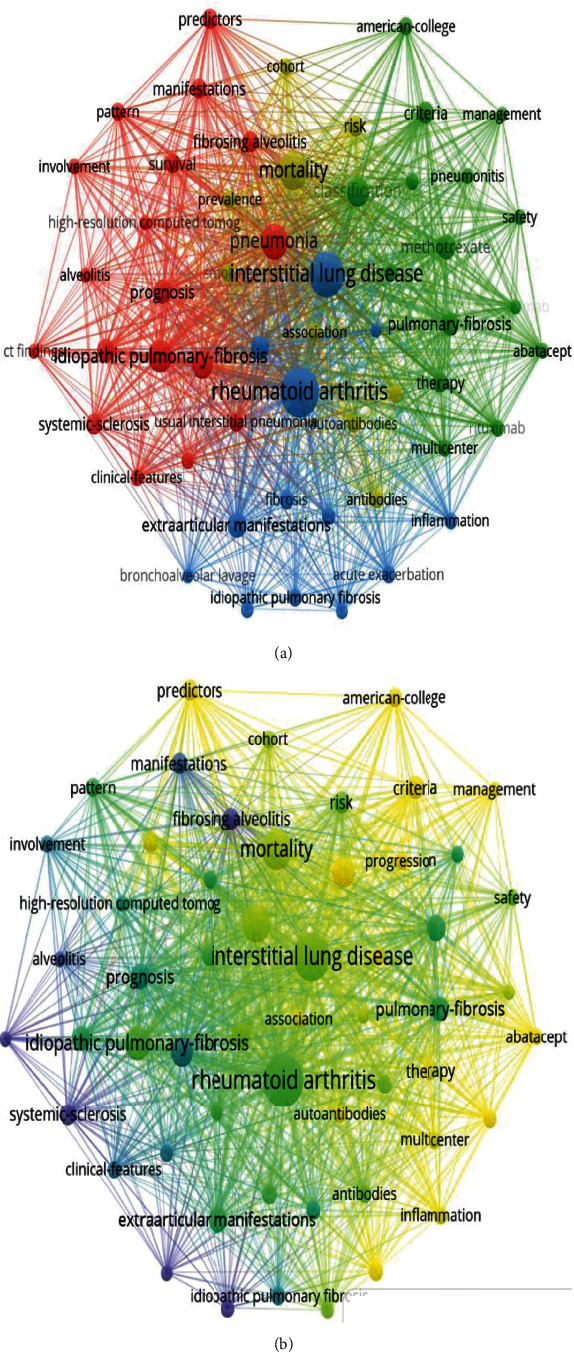
Keyword analysis. (a) Cluster analysis of keywords. (b) Evolution of keyword frequency.

VOSviewer software was used to analyze the cooccurrence analysis of keywords in the RA-ILD research articles. When the minimum number of keywords appearing in the publication was set to five, 72 keywords were selected and divided into four clusters:

“Clinical-Features,” “Pathological-Features,” “Treatment,” and “Prevalence and mortality.” In the “Clinical-Features” cluster, the most common keywords were “pneumonia,” “idiopathic pulmonary-fibrosis,” and “prognosis.” In the “Pathological-Features” cluster, the most frequent keywords were “rheumatoid arthritis,” “interstitial lung disease,” and “fibrosis.” In the “treatment” cluster, the most frequent keywords were “classification,” “criteria,” and “safety.” In the “prevalence and mortality” cluster, the most frequent keywords were “prevalence,” “mortality,” and “risk” (Figure 3(a)).

To better understand the dynamic process of the RA-ILD research trends, we evaluated the evolution of the keywords (Figure 3(b)). We assigned colors based on the year the keyword appears in the article. For example, the yellow keyword appears later than the purple keyword. In the early stages, “idiopathic pulmonary fibrosis,” “alveolitis,” and “systemic sclerosis” were the main topics. Trends in recent years show that the terms “management,” “predictors,” “inflammation,” and “progress” are becoming more and more popular.

### 3.6. The 100 Most-Cited Publications

**Figure 4:**
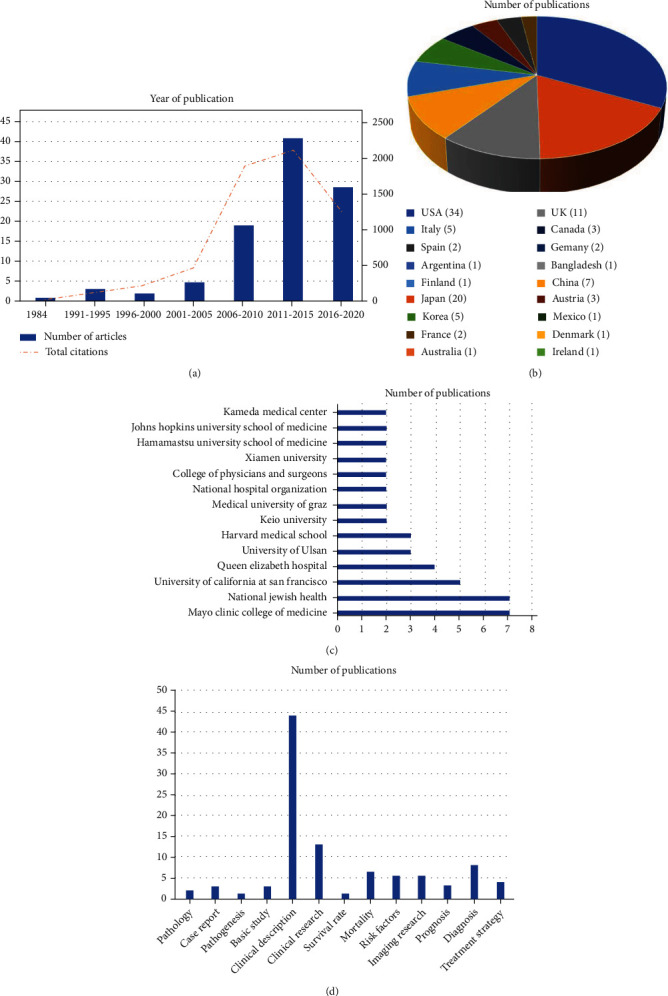
Top 100 most-cited publications on RA-ILD. (a) Year of publication. (b) Distribution of country. (c) Institution analysis. (e) Publication topics.

The 100 most-cited publications on RA-ILD were published between 1984 and 2020 (Table 2). The analysis indicated that 2001-2005 was the period when most of these studies were published, with 41 publications, followed by 2016-2020, with 29 publications (Figure 4(a)).

The 100 most-cited articles were from 18 countries and regions. Thirty-four articles were published by authors from the USA, followed by Japan (*n* = 20), the United Kingdom (*n* = 11), China (*n* = 7), Italy (*n* = 5), Canada (*n* = 3), Austria (*n* = 3), Spain, France, and Germany (*n* = 2), and Mexico, Argentina, Australia, Bangladesh, Denmark, Finland, and Ireland (*n* = 1) (Figure 4(b)).

Of these 100 articles, the Mayo Clinic Medicine and National Jewish Health each generated seven publications, resulting in their being the most represented institutions on this topic, followed by the University of California in San Francisco (*n* = 5) and Queen Elizabeth Hospital (*n* = 4) (Figure 4(c)).

Overall, there were 52 different journals which published the 100 articles. “Rheumatology” was the most productive journal, with 8 articles and 632 citations, followed by “Arthritis and Rheumatism,” with five articles and 529 citations (Table 3).

When considering the individual authors' academic contributions, Jay H Ryu, provided 11 publications, followed by Joyce C Lee and Eric L Matteson, each with 8 publications (Table 4).

The most common research topic on RA-ILD addressed the clinical description (*n* = 44), followed by clinical research (*n* = 13), diagnosis (*n* = 8), mortality (*n* = 7), and risk factors (*n* = 6) (Figure 4(d)).

## 4. Discussion

ILD is one of the most common complications of RA and poses a great challenge to clinicians and researchers [16]. The prevalence of RA-ILD ranged from 1% to 58% in the different studies, which was related to the diagnostic techniques used and the study population that was included [17–19]. According to the literature, there are many risk factors for RA-ILD, including male sex, smoking, older age, high disease activity of RA, characteristics of extra-articular diseases (subcutaneous nodules), and seropositive RA autoantibodies (rheumatoid factor and anticitrulline protein antibody) [2, 20–23]. The most common presenting symptoms include exertional dyspnea, tachypnea, and bibasilar inspiratory crackles. In the advanced stages of the disease, symptoms of cyanosis, edema, and pulmonary hypertension may occur, leading to a reduced quality of life [24].

In addition to its impact on the quality of life, RA-ILD places a huge burden on the medical system, with an average total medical cost of more than $170,000 per patient over five years [8]. Our statistical and quantitative analysis shows a gradual increase in RA-ILD research results from 2011 to 2020, with more researchers and physicians focusing on this area of research. Despite the wide range of RA-ILD research, an analysis of the current status and trends in RA-ILD research is not clear. In this study, we analyzed, discussed, and described the current status, priorities, and trends of RA-ILD research. At the same time, our study will help RA-ILD researchers gain a more comprehensive understanding of the current state of RA-ILD research and thus guide the direction of future research.

### 4.1. Publication Trends in RA-ILD Research

The number of articles related to RA-ILD has increased rapidly over the last 10 years. Globally, the USA ranks first in terms of the number of publications and citations, indicating that the USA has led to research on RA-ILD in the past few years. In terms of institutional contributions, the institution with the highest publication output is the Mayo Clinic (USA) and ranked first in the total citations. This reflects the institution's leadership in the field of RA-ILD research. Analysis of cooperation between countries and institutions shows that regional clusters are usually geographically specific. As a leader in the world economy and science, the USA has the most frequent cooperation with Japan, France, China, and South Korea. Researchers working on RA-ILD should pay close attention to them and collaborate with these institutions and countries. Annals of the Rheumatic Diseases, Arthritis & Rheumatology, American Journal of Respiratory and Critical Care Medicine, Rheumatology, and Arthritis and Rheumatism are the five most prolific journals in RA-ILD.

### 4.2. Research Foci

Keyword analysis results showed that RA-ILD, rheumatoid arthritis, interstitial lung disease, and pneumonia were keyword cluster centers. In the early stages, “idiopathic pulmonary fibrosis,” “alveolitis,” and “systemic sclerosis” were the main topics. In recent years, more common keywords have included “management,” “predictors,” “inflammation,” and “progression.”

### 4.3. The Most-Cited Articles

The most-cited publication in RA-ILD was the 2010 article in Arthritis and Rheumatism by Bongartz et al. with 324 citations: “Incidence and mortality of interstitial lung disease in rheumatoid arthritis: a population-based study,” which introduced incidence, risk factors, and mortality of RA-ILD [2]. The mean follow-up time of 582 RA patients and 603 non-RA patients was 16.4 years and 19.3 years, respectively. The lifetime risk of ILD was 7.7% in patients with rheumatoid arthritis and 0.9% in those without rheumatoid arthritis. Studies have shown that the prevalence of ILD is higher in older male patients and in individuals with more severe RA parameters. RA patients diagnosed with ILD have poorer survival than RA patients without ILD, and ILD accounts for approximately 13% of the excess mortality in RA patients compared to the general population.

“Usual interstitial pneumonia in rheumatoid arthritis-associated interstitial lung disease” by Kim et al. in 2010 was the second most-cited article with 283 citations [16]. The authors determined that the pattern of common interstitial pneumonia (IP) found on high-resolution computed tomography (HRCT) is important for the prognosis of RA-ILD. Eighty-two patients with RA-ILD were identified retrospectively. “We determined the relationship between survival and the pattern of IP common on HRCT and compared it with patients diagnosed radiologically with idiopathic pulmonary fibrosis. Twenty (24%) of the 82 patients with RA-ILD had definite common IP. Survival in patients with RA-ILD was lower than that in patients without this pattern, similar to the survival of patients with idiopathic pulmonary fibrosis. In addition, a clear pattern of common IP on HRCT was associated with poor survival. Analysis of feature-specific HRCTs showed that traction bronchiectasis and cellular fibrosis were associated with poor survival. Women and a higher baseline carbon monoxide lung diffusing capacity were associated with better survival.”

“Histopathological and clinical features of interstitial lung disease associated with rheumatoid arthritis” by Lee et al. was the third most-cited article with 245 citations [25]. The authors studied the histopathological patterns and clinical characteristics of patients with RA-ILD according to the American Thoracic Society/European Respiratory Society consensus classification of idiopathic IP. “Eighteen patients with RA who underwent surgical lung biopsy for suspected ILD were included in this study. This study revealed diverse histopathological findings. Ten patients had a common interstitial pneumonia (UIP) pattern, six patients had a nonspecific interstitial pneumonia (NSIP) pattern, and two patients had inflammatory airway disease with tissue-type pneumonia. Thus, the UIP pattern appears to be more common than the NSIP pattern in our study population.”

### 4.4. Limitations

Our study had several limitations. First, we extracted information related to RA-ILD from the Core Collection database of the Web of Science. It is possible that some influential publications were not included in this database and were therefore excluded from our study. Second, the date of our retrieval and extraction of data was July 1, 2021. Part of the data correspond to dynamic changes, but the trend of changes will not be extensive. Third, we retained only English articles in our search strategy.

## 5. Conclusions

Quantitative analysis showed that in the past 10 years, global research on RA-ILD has increased rapidly. Of all the countries, the USA publishes most articles on RA-ILD.

The USA has contributed the most to the RA-ILD literature. Mayo Clinic, National Jewish Health, Brigham and Women's Hospital, Colorado State University, and University of California, San Francisco are the most prolific institutions associated with RA-ILD research. Annals of the Rheumatic Diseases, Arthritis & Rheumatology, American Journal of Respiratory and Critical Care Medicine, Rheumatology, and Arthritis and Rheumatism are the top five most popular journals on RA-ILD publications.

## Figures and Tables

**Table 1:** Active journals on rheumatoid arthritis-associated interstitial lung disease.

Journal	Article	Total citation	Mean citation	Impact factor
Annals of the Rheumatic Diseases	93	338	3.63	19.103
Arthritis & Rheumatology	75	219	2.92	10.995
American Journal of Respiratory and Critical Care Medicine	53	514	9.70	21.405
Rheumatology	38	710	18.68	20.543
Arthritis and Rheumatism	36	552	15.33	5.532
European Respiratory Journal	30	537	17.90	16.671
Clinical Rheumatology	18	248	13.78	4.098
Chest	12	485	40.42	2.878
Internal Medicine	11	156	14.18	2.048
Clinical and Experimental Rheumatology	10	98	9.80	4.473
Respiratory Medicine	8	258	32.25	3.772
Modern Rheumatology	8	108	13.50	3.023
Seminars in Arthritis and Rheumatism	6	208	34.67	5.532
Rheumatology International	6	154	25.67	2.631
Journal of Rheumatology	6	147	24.50	4.666
New England Journal of Medicine	6	133	22.17	91.245
PLos one	6	127	21.17	3.24
Scandinavian Journal of Rheumatology	6	104	17.33	3.641
Respirology	6	92	15.33	6.424
JCR-Journal of Clinical Rheumatology	6	26	4.33	3.517

**Table 2:** The top 100 cited publications on rheumatoid arthritis-associated interstitial lung disease.

Rank	Title	Author	Journal	Year	Total citation	Citation/year
1	Incidence and mortality of interstitial lung disease in rheumatoid arthritis: a population-based study	Bongartz, T.	Arthritis and Rheumatism	2010	324	27
2	Usual interstitial pneumonia in rheumatoid arthritis-associated interstitial lung disease	Kim, E. J.	European Respiratory Journal	2010	283	23.58
3	Histopathologic pattern and clinical features of rheumatoid arthritis associated interstitial lung disease	Lee, H. K	Chest	2005	245	14.41
4	Rheumatoid arthritis-interstitial lung disease-associated mortality	Olson, A. L.	American Journal of Respiratory and Critical Care Medicine	2011	222	20.18
5	Interstitial lung disease in recent onset rheumatoid arthritis	Gabbay, E.	American Journal of Respiratory and Critical Care Medicine	1997	220	8.8
6	Rheumatoid arthritis-related interstitial lung disease: associations, prognostic factors and physiological and radiological characteristics-a large multicentre UK study	Kelly, C. A.	Rheumatology	2014	205	25.63
7	Progressive preclinical interstitial lung disease in rheumatoid arthritis	Gochuico, B. R.	Archives of Internal Medicine	2008	198	14.14
8	Rheumatoid arthritis-associated interstitial lung disease the relevance of histopathologic and radiographic pattern	Kim, E. J.	Chest	2009	189	14.54
9	Interstitial lung disease has a poor prognosis in rheumatoid arthritis: results from an inception cohort	Koduri, G.	Rheumatology	2010	149	12.42
10	Interstitial lung diseases induced or exacerbated by DMARDS and biologic agents in rheumatoid arthritis: A systematic literature review	Roubille, C.	Seminars in Arthritis and Rheumatism	2014	126	15.75
11	Predictors of mortality in rheumatoid arthritis-associated interstitial lung disease	Solomon, J. J.	European Respiratory Journal	2016	124	20.67
12	MUC5B promoter variant and rheumatoid arthritis with interstitial lung disease	Juge, P. A.	New England Journal of Medicine	2018	108	27
13	Influence of anti-TNF therapy on mortality in patients with rheumatoid arthritis-associated interstitial lung disease: results from the British Society for Rheumatology Biologics Register	Dixon, W. G.	Annals of the Rheumatic Diseases	2010	108	9
14	High-resolution computed-tomography of the lungs in patients with rheumatoid-arthritis and interstitial lung-disease	Mcdonagh, J	British Journal of Rheumatology	1994	102	3.64
15	Leflunomide use and the risk of interstitial lung disease in rheumatoid arthritis	Suissa, S.	Arthritis and Rheumatism	2006	95	5.94
16	Different risk factors between interstitial lung disease and airway disease in rheumatoid arthritis	Mori, S.	Respiratory Medicine	2012	89	8.9
17	A population-based cohort study of rheumatoid arthritis-associated interstitial lung disease: comorbidity and mortality	Hyldgaard, C.	Annals of the Rheumatic Diseases	2017	83	16.6
18	Predictors of mortality in rheumatoid arthritis-related interstitial lung disease	Assayag, D.	Respirology	2014	83	10.38
19	Rheumatoid arthritis-associated interstitial lung disease: radiologic identification of usual interstitial pneumonia pattern	Assayag, D.	Radiology	2014	77	9.63
20	Correlation between HRCT findings, pulmonary function tests and bronchoalveolar lavage cytology in interstitial lung disease associated with rheumatoid arthritis	Biederer, J.	European Radiology	2004	74	4.11
21	Association of fine specificity and repertoire expansion of anticitrullinated peptide antibodies with rheumatoid arthritis associated interstitial lung disease	Giles, J. T.	Annals of the Rheumatic Diseases	2014	71	8.88
22	Fibrosing interstitial pneumonia predicts survival in patients with rheumatoid arthritis-associated interstitial lung disease (RA-ILD)	Solomon, J. J.	Respiratory Medicine	2013	71	7.89
23	Effect of rituximab on the progression of rheumatoid arthritis-related interstitial lung disease: 10 years' experience at a single centre	Yusof, M. M.	Rheumatology	2017	69	13.8
24	Shared genetic predisposition in rheumatoid arthritis-interstitial lung disease and familial pulmonary fibrosis	Juge, P. A.	European Respiratory Journal	2017	69	13.8
25	Rheumatoid arthritis (RA)-specific autoantibodies in patients with interstitial lung disease and absence of clinically apparent articular RA	Gizinski, A. M.	Clinical Rheumatology	2009	68	5.23
26	Detection of rheumatoid arthritis-interstitial lung disease is enhanced by serum biomarkers	Doyle, T. J.	American Journal of Respiratory and Critical Care Medicine	2015	67	9.57
27	Leflunomide-induced interstitial lung disease: prevalence and risk factors in Japanese patients with rheumatoid arthritis	Sawada, T.	Rheumatology	2009	66	5.08
28	Acute exacerbation in rheumatoid arthritis-associated interstitial lung disease: a retrospective case control study	Hozumi, H.	BMJ open	2013	63	7
29	The lung in rheumatoid arthritis focus on interstitial lung disease	Spagnolo, P.	Arthritis & Rheumatology	2018	62	15.5
30	Abatacept in patients with rheumatoid arthritis and interstitial lung disease: a national multicenter study of 63 patients	Fernandez-Diaz, C.	Seminars in Arthritis and Rheumatism	2018	62	15.5
31	Rheumatoid arthritis (RA) associated interstitial lung disease (ILD)	O'Dwyer, D. N.	European Journal of Internal Medicine	2013	62	6.89
32	Rheumatoid arthritis treatment and the risk of severe interstitial lung disease	Wolfe, F.	Scandinavian Journal of Rheumatology	2007	60	4
33	Rheumatoid arthritis complicated with acute interstitial pneumonia induced by leflunomide as an adverse reaction	Kamata, Y	Internal Medicine	2004	56	3.11
34	Morphologic and quantitative assessment of CD20+ B cell infiltrates in rheumatoid arthritis-associated nonspecific interstitial pneumonia and usual interstitial pneumonia	Atkins, S. R.	Arthritis and Rheumatism	2006	55	3.44
35	Biomarkers of rheumatoid arthritis-associated interstitial lung disease	Chen, J.	Arthritis & Rheumatology	2015	54	7.71
36	Acute exacerbation of preexisting interstitial lung disease after administration of etanercept for rheumatoid arthritis	Hagiwara, K.	Journal of Rheumatology	2007	52	3.47
37	Nonspecific interstitial pneumonia pattern as pulmonary involvement of rheumatoid arthritis	Yoshinouchi, T	Rheumatology International	2005	49	2.88
38	Progressive decline of lung function in rheumatoid arthritis-associated interstitial lung disease	Zamora-Legoff, J. A.	Arthritis & Rheumatology	2017	48	9.6
39	Retrospective study of the clinical characteristics and risk factors of rheumatoid arthritis-associated interstitial lung disease	Zhang, Y. F.	Clinical Rheumatology	2017	46	9.2
40	Rheumatoid arthritis-interstitial lung disease in the United States: prevalence, incidence, and healthcare costs and mortality	Raimundo, K.	Journal of Rheumatology	2019	45	15
41	The multifaceted aspects of interstitial lung disease in rheumatoid arthritis	Cavagna, L.	Biomed Research International	2013	45	5
42	Association of human leukocyte antigen with interstitial lung disease in rheumatoid arthritis: a protective role for shared epitope	Furukawa, H.	PLos one	2012	45	4.5
43	Clinical and radiological features of acute-onset diffuse interstitial lung diseases in patients with rheumatoid arthritis receiving treatment with biological agents: importance of Pneu	Kameda, H.	Internal Medicine	2011	45	4.09
44	Is incident rheumatoid arthritis interstitial lung disease associated with methotrexate treatment? Results from a multivariate analysis in the ERAS and ERAN inception cohorts	Kiely, P.	BMJ open	2019	44	14.67
45	High resolution computed tomography pattern of usual interstitial pneumonia in rheumatoid arthritis-associated interstitial lung disease: relationship to survival	Yunt, Z. X.	Respiratory Medicine	2017	44	8.8
46	Interstitial lung disease in rheumatoid arthritis: recent advances	Kim, D. S.	Current Opinion in Pulmonary Medicine	2006	44	2.75
47	Treatment of rheumatoid arthritis-associated interstitial lung disease: a perspective review	Iqbal, K.	Therapeutic Advances in Musculoskeletal Disease	2015	43	6.14
48	Clinical course and outcome of rheumatoid arthritis-related usual interstitial pneumonia	Song, J. W.	Sarcoidosis Vasculitis and Diffuse Lung Diseases	2013	42	4.67
49	Increased levels of interleukin-33 associated with bone erosion and interstitial lung diseases in patients with rheumatoid arthritis	Zhu X. Y	Cytokine	2012	42	4.2
50	Incidence of and risk factors for interstitial pneumonia in patients with rheumatoid arthritis in a large Japanese observational cohort, IORRA	Shidara, K.	Modern Rheumatology	2010	42	3.5
51	Interstitial lung-disease in rheumatoid-arthritis - assessment with high-resolution computed-tomography	Fujii, M	Journal of Thoracic Imaging	1993	42	1.45
52	Patterns of interstitial lung disease and mortality in rheumatoid arthritis	Zamora-Legoff, J. A.	Rheumatology	2017	41	8.2
53	Standard and pocket-size lung ultrasound devices can detect interstitial lung disease in rheumatoid arthritis patients	Cogliati, C.	Rheumatology	2014	41	5.13
54	Potential risk of TNF inhibitors on the progression of interstitial lung disease in patients with rheumatoid arthritis	Nakashita, T.	BMJ open	2014	41	5.13
55	A fatal case of acute exacerbation of interstitial lung disease in a patient with rheumatoid arthritis during treatment with tocilizumab	Kawashiri, S.	Rheumatology International	2012	41	4.1
56	Interstitial lung disease in patients with rheumatoid arthritis: comparison with cryptogenic fibrosing alveolitis over 5 years	Rajasekaran, A.	Journal of Rheumatology	2006	41	2.56
57	Interstitial lung disease in patients with rheumatoid arthritis: a comparison with cryptogenic fibrosing alveolitis	Rajasekaran, B. A.	Rheumatology	2001	40	1.9
58	A roadmap to promote clinical and translational research in rheumatoid arthritis-associated interstitial lung disease a dance promote clinical and translational research	Doyle, T. J.	Chest	2014	39	4.88
59	Rheumatoid arthritis-related interstitial lung disease (RA-ILD): methotrexate and the severity of lung disease are associated to prognosis	Rojas-serrano, J.	Clinical Rheumatology	2017	38	7.6
60	Rheumatoid arthritis associated interstitial lung disease: a review	Assayag, D.	Medicina-Buenos Aires	2014	38	4.75
61	Sonographic assessment of interstitial lung disease in patients with rheumatoid arthritis, systemic sclerosis and systemic lupus erythematosus	Moazedi-Fuerst, F.	Clinical and Experimental Rheumatology	2015	37	5.29
62	Association of cross-reactive antibodies targeting peptidyl-arginine deiminase 3 and 4 with rheumatoid arthritis-associated interstitial lung disease	Giles, J. T.	PLos one	2014	37	4.63
63	Clinical and laboratory factors associated with interstitial lung disease in rheumatoid arthritis	Restrepo, J. F.	Clinical Rheumatology	2015	35	5
64	Rheumatoid arthritis interstitial lung disease: mycophenolate mofetil as an antifibrotic and disease-modifying antirheumatic drug	Saketkoo, L. A.	Archives of Internal Medicine	2008	34	2.43
65	Survival and quality of life in rheumatoid arthritis-associated interstitial lung disease after lung transplantation	Yazdani, A.	Journal of Heart and Lung Transplantation	2014	33	4.13
66	Rheumatoid arthritis-associated autoantibodies and subclinical interstitial lung disease: the multi-ethnic study of atherosclerosis	Bernstein, E. J.	Thorax	2016	32	5.33
67	Nintedanib reduces pulmonary fibrosis in a model of rheumatoid arthritis-associated interstitial lung disease	Redente, E. F.	American Journal of Physiology-Lung Cellular and Molecular phy	2018	31	7.75
68	Profibrotic effect of IL-17A and elevated IL-17RA in idiopathic pulmonary fibrosis and rheumatoid arthritis-associated lung disease support a direct role for IL-17A/IL-17RA in human fib	Zhang, J.	American Journal of Physiology-Lung Cellular and Molecular phy	2019	30	10
69	Variable course of disease of rheumatoid arthritis-associated usual interstitial pneumonia compared to other subtypes	Nurmi, H. M.	BMC Pulmonary Medicine	2016	30	5
70	Risk of interstitial lung disease associated with leflunomide treatment in Korean patients with rheumatoid arthritis	Ju, J. H.	Arthritis and Rheumatism	2007	30	2
71	Anti-cyclic citrullinated peptide antibody is associated with interstitial lung disease in patients with rheumatoid arthritis	Yin, Y. F.	PLos one	2014	29	3.63
72	A novel model of rheumatoid arthritis-associated interstitial lung disease in SKG mice	Keith, R. C.	Experimental Lung Research	2012	29	2.9
73	Treatment strategies for a rheumatoid arthritis patient with interstitial lung disease	Kelly, C.	Expert Opinion on Pharmacotherapy	2008	28	2
74	Rheumatoid arthritis disease activity predicting incident clinically apparent rheumatoid arthritis-associated interstitial lung disease: a prospective cohort study	Sparks, J. A.	Arthritis & Rheumatology	2019	27	9
75	Changes in peripheral CD19(+) Foxp3(+) and CD19(+) TGF beta(+) regulatory B cell populations in rheumatoid arthritis patients with interstitial lung disease	Guo, Y. Y.	Journal of Thoracic Disease	2015	27	3.86
76	Interstitial lung disease in patients with rheumatoid arthritis: spontaneous and drug induced	Hallowell, R. W.	Drugs	2014	27	3.38
77	HLA-A∗31 : 01 and methotrexate-induced interstitial lung disease in Japanese rheumatoid arthritis patients: a multidrug hypersensitivity marker?	Furukawa, H.	Annals of the Rheumatic Diseases	2013	27	3
78	Asymptomatic preclinical rheumatoid arthritis-associated interstitial lung disease	Chen, J.	Clinical & Developmental Immunology	2013	27	3
79	Rheumatoid arthritis-associated interstitial lung disease and idiopathic pulmonary fibrosis: shared mechanistic and phenotypic traits suggest overlapping disease mechanisms	Paulin, F.	Revista de Investigacion Clinica-Clinical and Translational Investig	2015	26	3.71
80	Lymphoid interstitial pneumonia in juvenile rheumatoid-arthritis	Lovell, D.	Journal of Pediatrics	1984	26	0.68
81	Ultrasound screening for interstitial lung disease in rheumatoid arthritis	Moazedi-Fuerst, F. C.	Clinical and Experimental Rheumatology	2014	25	3.13
82	Interstitial pneumonia due to cytomegalovirus following low-dose methotrexate treatment for rheumatoid-arthritis	Aglas, F.	Arthritis and Rheumatism	1995	25	0.93
83	Therapeutic management of patients with rheumatoid arthritis and associated interstitial lung disease: case report and literature review	Diamanti, A. P.	Therapeutic Advances in Respiratory Disease	2017	24	4.8
84	Interstitial lung disease in rheumatoid arthritis: response to IL-6R blockade	Mohr, M.	Scandinavian Journal of Rheumatology	2011	24	2.18
85	Tocilizumab therapy in rheumatoid arthritis with interstitial lung disease: a multicentre retrospective study	Manfredi, A.	Internal Medicine Journal	2020	23	11.5
86	Recent advances in the pathogenesis, prediction, and management of rheumatoid arthritis-associated interstitial lung disease	Johnson, C.	Current Opinion in Rheumatology	2017	23	4.6
87	Abatacept therapy in rheumatoid arthritis with interstitial lung disease	Mera-Varela, A.	Journal of Clinical Rheumatology	2014	23	2.88
88	The clinical significance of HRCT in evaluation of patients with rheumatoid arthritis-associated interstitial lung disease: a report from China	Zou, Y. Q.	Rheumatology International	2012	23	2.3
89	A case of adalimumab-associated interstitial pneumonia with rheumatoid arthritis	Yamazaki, H.	Modern Rheumatology	2010	23	1.92
90	Prevalence and effects of emphysema in never-smokers with rheumatoid arthritis interstitial lung disease	Jacob, J.	Ebiomedicine	2018	22	5.5
91	Association of disease activity with acute exacerbation of interstitial lung disease during tocilizumab treatment in patients with rheumatoid arthritis: a retrospective, case-control study	Akiyama, M.	Rheumatology International	2016	22	3.67
92	Predicting outcomes in rheumatoid arthritis related interstitial lung disease	Jacobt, J.	European Respiratory Journal	2019	21	7
93	Plasma miRNA expression profiles in rheumatoid arthritis associated interstitial lung disease	Oka, S.	BMC Musculoskeletal Disorders	2017	21	4.2
94	Patients with limited rheumatoid arthritis-related interstitial lung disease have a better prognosis than those with extensive disease	Sathi, N.	Rheumatology	2011	21	1.91
95	Risk of serious infection in patients with rheumatoid arthritis-associated interstitial lung disease	Zamora-Legoff, J. A.	Clinical Rheumatology	2016	20	3.33
96	Possible effect of abatacept on the progression of interstitial lung disease in rheumatoid arthritis patients	Nakashita, T.	Respiratory Investigation	2016	20	3.33
97	Up-to-date information on rheumatoid arthritis-associated interstitial lung disease	Suda, T.	Clinical Medicine Insights-Circulatory Respiratory and Pulmonary	2015	20	2.86
98	Eternacept for the treatment of patients with rheumatoid arthritis and concurrent interstitial lung disease	Horai, Y.	Journal of Clinical Pharmacy and Therapeutics	2012	20	2
99	Myofibroblasts and S-100 protein positive cells in idiopathic pulmonary fibrosis and rheumatoid arthritis-associated interstitial pneumonia	Yoshinouchi, T.	European Respiratory Journal	1999	20	0.87
100	The performance of the GAP model in patients with rheumatoid arthritis associated interstitial lung disease	Morisset, J.	Respiratory Medicine	2017	19	3.8

**Table 3:** Journal with more than three of the 100 most-cited publications on rheumatoid arthritis-associated interstitial lung disease.

Journal	Article	Total citation	Mean citation	Impact factor
Rheumatology	8	632	79	3.494
Arthritis and Rheumatism	5	529	105.8	5.532
Clinical Rheumatology	5	207	41.4	4.098
European Respiratory Journal	5	517	103.4	16.671
Annals of the Rheumatic Diseases	4	289	72.25	19.103
Arthritis & Rheumatology	4	191	47.75	10.995
Respiratory Medicine	4	223	55.75	3.772
Rheumatology International	4	135	33.75	2.631

**Table 4:** Most frequent authors of the 100 most-cited publications on rheumatoid arthritis-associated interstitial lung disease.

Author	Article	First author	Last author	Co-author
Ryu, Jay H.	11	0	0	0
Lee, Joyce S.	8	0	2	6
Matteson, Eric L.	8	2	4	2
Brown, Kevin K.	6	0	2	4
Collard, Harold R.	6	0	1	5
Kelly, Clive A.	6	2	4	0
Kim, Dong Soon.	6	1	1	4
Rosas, Ivan O.	6	0	3	3
Solomon, Joshua J.	6	2	1	3
Doyle, Tracy J.	5	2	1	1
Ascherman, Dana P.	5	0	2	3
Fischer, Aryeh.	5	0	0	0
Swigris, Jeffrey J.	5	0	2	3
